# Melanose der Harnblase – eine Rarität

**DOI:** 10.1007/s00120-023-02127-z

**Published:** 2023-06-27

**Authors:** S. Hagmann, D. Born, H. P. Schmid, J. Rührup

**Affiliations:** 1 Klinik für Urologie, Kantonsspital St. Gallen, Rorschacher Str. 95, 9007 St. Gallen, Schweiz; 2 Institut für Pathologie, Kantonsspital St. Gallen, St. Gallen, Schweiz

**Keywords:** Melanose, Harnblase, Neurogene Blasenentleerungsstörung, Drangsymptomatik, Zystoskopie, Melanosis, Bladder, Neurogenic voiding dysfunction, Urgency, Cystoscopy

## Abstract

Die Melanose der Harnblase ist eine äußerst seltene, gutartige Erscheinung, bei der es zu Melaninablagerungen in den Urothel- und Stromazellen kommt. Wir berichten über einen solchen Fall, bei dem im Rahmen einer erweiterten Abklärung aufgrund einer Drangsymptomatik bei einer 55-jährigen Patientin mit bekannter multipler Sklerose eine Melanose der Harnblase festgestellt wurde. Der Befund wurde mittels einer Biopsie bestätigt.

## Abstract (en)

Melanosis of the urinary bladder is an extremely rare benign condition in which melanin deposits occur in the urothelial and stromal cells. We report such a case in which melanosis of the urinary bladder was detected in a 55-year-old woman with known multiple sclerosis during an extended workup due to urinary urgency complaints. The findings were confirmed by biopsy.

## Falldarstellung

### Anamnese

Die 55-jährige Patientin stellte sich bei Verdacht auf eine neurogene Blasenentleerungsstörung im Rahmen einer bekannten multiplen Sklerose (MS) zur weiterführenden urodynamischen Abklärung in unserer urologischen Klinik vor.

Seit mehreren Jahren lag eine Drangsymptomatik vor, welche seit August 2020 mittels eines β_3_-Sympathomimetikums (Mirabegron) therapiert wurde. Hierunter kam es jedoch nur zu einer ungenügenden Symptomlinderung der Urgesymptomatik. Die Drangbeschwerden zeigten sich im Verlauf so ausgeprägt, dass die Patientin zum Zeitpunkt der Vorstellung in unserer Klinik ihren Tagesablauf an die Erreichbarkeit einer öffentlichen Toilette anpassen musste. Zu Urinverlust im Sinne einer Dranginkontinenz sei es nur selten und tröpfchenweise gekommen, eine Belastungsinkontinenz lag nicht vor. Bis vor 5 Jahren sei es gehäuft zu Antibiotika-pflichtigen Zystitiden gekommen, welche seither nicht mehr aufgetreten seien. Eine Vakzinierung ist nicht erfolgt.

Nebst der bekannten MS mit schubförmiger Verlaufsform und Übergang in die sekundäre Progredienz besteht außerdem ein St. n. schwerem Schädel-Hirn-Trauma nach Treppensturz im November 2018 und ein Vitamin-D-Mangel ohne weitere Vorerkrankungen. Die Patientin ist Nichtraucherin. Die Familienanamnese ist blande bezüglich urologischer oder dermatologischer Neoplasien und relevanter Vorerkrankungen.

Die Patientin ist unter regelmäßiger Medikation mit Fingolimod, Mirabegron, Escitalopram und Alpha-D-Mannose.

### Befund

Die Patientin präsentiert sich bei Vorstellung in einem aufgrund der vorbestehenden MS leicht reduzierten Allgemeinzustand. Sie ist Rollator-mobil und voll orientiert.

Im Miktionsprotokoll zeigten sich Urinportionen zwischen 100 ml bis maximal 280 ml. Die Trinkmenge belief sich auf 1,5 l pro Tag. Es bestand eine tägliche Miktion von ca. 7 × und eine Nykturie von meist 1–2 ×.

### Diagnose

Zur weiteren Abklärung der Drangsymptomatik wurden eine Sonographie, eine Manometrie und eine Zystoskopie durchgeführt. Sonographisch zeigten sich die Nieren beidseits mit unauffälligem Parenchym mit kleinen Nierenzysten beidseits ohne Dilatation des Nierenbeckenkelchsystems und ohne darstellbare Konkremente. Die Harnblase stellte sich sonographisch ebenfalls unauffällig und glatt berandet dar, es bestand eine Restharnbildung von 100 ml.

In der zur weiteren Diagnostik durchgeführten Manometrie zeigte sich eine hypokapazitive, hypersensitive, hypokontraktile Harnblase mit terminal instabilem Detrusor mit Detrusor-Sphinkter-Dyskoordination sowie formal erniedrigter Compliance. Es lag eine Gefährdung des oberen Harntrakts vor bei Detrusordrücken bis 63 cm H_2_O.

**Figure 1:**
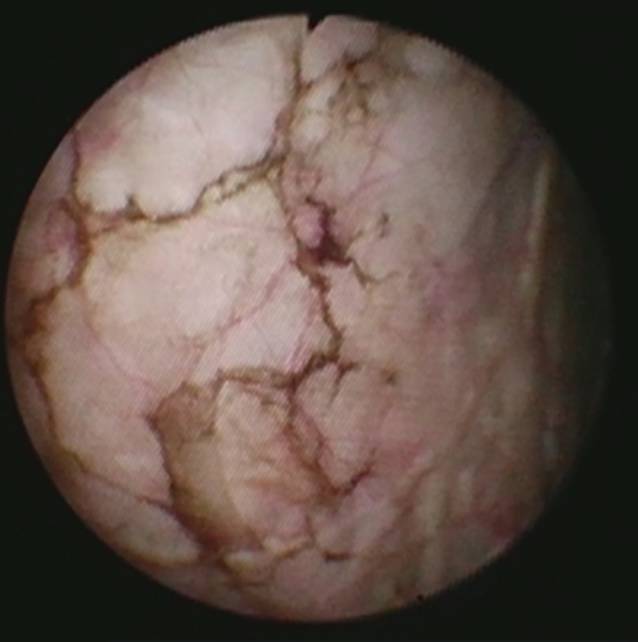

Zystoskopisch zeigte sich eine unauffällige, nicht obstruktiv wirkende Harnröhre und eine leicht trabekularisierte Harnblase. Ubiquitär im Bereich der Blasenwand (v. a. verstärkt an der Blasenseitenwand links am Übergang zur Blasenhinterwand sowie an der Blasenwand rechts) zeigte sich eine feine bräunliche, nicht erhabene, netzartige Zeichnung. Die Ostien waren hiervon nicht affektiert und normwertig konfiguriert und es zeigte sich kein exophytisches oder papilläres Wachstum intravesikal (Abb. 1).

Die entnommene Urinzytologie zeigte Urothelzellen mit z. T. grobkörnigem braungrauem Material im Zytoplasma. Die körnigen Ablagerungen im Zytoplasma der Urothelzellen konnten nicht weiter typisiert werden. Postuliert wurden Lipofuszin-Ablagerungen. Maligne Zellen konnten nicht beobachtet werden.

### Therapie und Verlauf

Aufgrund der oben genannten urodynamischen Befunde wurde eine Beckenbodenphysiotherapie mit Biofeedback sowie eine ergänzende anticholinerge Therapie mit Trospium chlorid zum Schutz der oberen Harnwege initiiert. Aufgrund der unklaren Befunde in der Zystoskopie wurde eine Biopsieentnahme mittels transurethraler Resektion durchgeführt. Postoperativ zeigten sich verstärkte irritative Miktionsbeschwerden und das Vorliegen einer Bakteriurie bei einem sonographischen Restharnvolumen von 110 ml, sodass bei Verdacht auf eine Harnwegsinfektion eine antibiotische Therapie mit Co-Amoxicillin für 5 Tage resistenzgerecht initiiert wurde.

### Histopathologie

**Figure 2:**
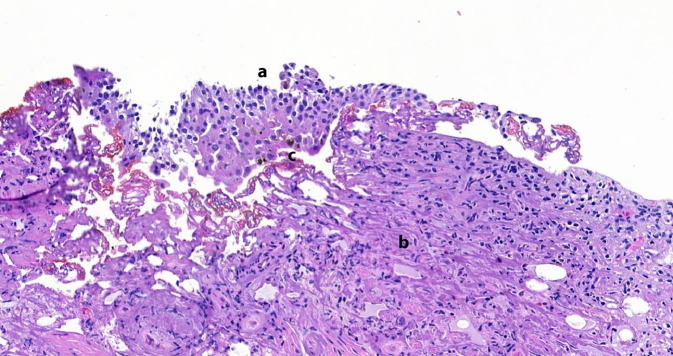

Der histopathologische Befund zeigte neben einer ausgedehnten chronischen Entzündung unter Beteiligung von eosinophilen Granulozyten immer wieder braunes granuläres Pigment sowohl in den Urothelzellen als auch in den stromalen Zellen. Dies konnte mittels einer zusätzlichen Melaninfärbung bestätigt werden. Dieser Befund passt sehr gut zu dem klinischen Bild einer Melanose der Harnblase. In den vorliegenden Biopsien wurden keine malignen Zellen beobachtet. Somit konnte eine Assoziation mit einem Malignom ausgeschlossen werden (Abb. 2 und 3).

**Figure 3:**
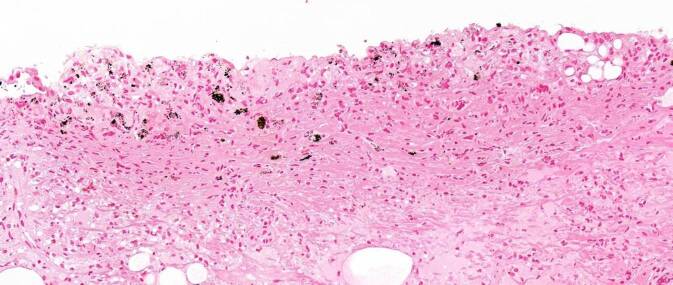

## Diskussion

Die Melanose der Harnblase ist eine sehr seltene, benigne Erscheinung, bei der es zu einer Ansammlung von Melanin im Urothel und Stroma kommt. In der Literatur sind weniger als 30 Fälle beschrieben, und eine Pathogenese konnte bislang nicht schlüssig eruiert werden [1]. Diagnostiziert wurden die Fälle meistens als Zufallsbefund im Rahmen weiterführender Diagnostik zur Abklärung von z. B. Drangbeschwerden – so auch in unserem Fall. Ob die Melanose Ursache von irritativen Beschwerden ist, ist mehrheitlich ungeklärt [2]. Somit ist schlussendlich nicht eindeutig zu evaluieren, ob die Drangsymptomatik unserer Patientin durch die Melanose mitbedingt ist. Insgesamt ist bezüglich der Melanose keine Therapie indiziert.

Es wurden wenige Fälle beschrieben, bei denen eine Melanose zusammen mit einem Melanom der Harnblase respektive einem Urothelkarzinom der Harnblase aufgetreten sind [3]. Ein kausaler Zusammenhang konnte jedoch bisher ebenfalls nicht festgestellt werden. Auch in unserem Falle konnte ein Melanom oder ein Urothelkarzinom histologisch ausgeschlossen werden. Dennoch ist der bioptische Ausschluss eines malignen Geschehens unumgänglich. Hierbei empfiehlt sich die Melaninfärbung zur histologischen Bestätigung von Melaninablagerungen. Eine genetische syndromale Assoziation wie bei anderen Schleimhautmelanosesituationen (z. B. Peutz-Jeghers-Syndrom) ist nicht bekannt. Guidelines zum Follow-up gibt es bisher keine, jedoch wird in einer Publikation von einer empfohlenen 1‑ bis 2‑jährlichen zystoskopischen Reevaluation gesprochen, aufgrund der möglichen Assoziation einer Melanosis vesicae mit malignem Melanom und Urothelkarzinom [4]. Im Gegensatz zur Melanose treten bei der Pseudomelanose Laxantien-assoziierte Lipofuszin-Ablagerungen auf, welche ebenfalls ein bräunliches Erscheinungsbild haben und hauptsächlich im Darm beschrieben werden.

Grundsätzlich ist bei jeglicher Form der Schleimhautmelanose eine Ganzhautinspektion empfohlen. Daher wurde auch in unserem Fallbeispiel zum Ausschluss eines Melanoms eine Ganzkörperhautinspektion durch die Dermatologie durchgeführt. Hierbei zeigte sich eine auffällige Papel mit zentralem Krater am linken Unterlid. Zudem fand sich ein „ugly duckling“ Nävus lumbal links, welcher exzidiert wurde. In der histologischen Untersuchung des Befunds am linken Augenlid zeigte sich ein Molluscum contagiosum ohne Anhaltspunkte für Malignität.

Urologischerseits sind jährliche zystoskopische Verlaufskontrollen geplant. Seitens der Kollegen der Dermatologie wird die Patientin ebenfalls für eine jährliche Ganzkörperinspektion aufgeboten.

## Fazit für die Praxis

- Bei unklarem Befund in der Zystoskopie ist zum Ausschluss eines malignen Geschehens immer eine bioptische Untersuchung indiziert.
- Eine interdisziplinäre Zusammenarbeit ist angezeigt um die Patientinnen optimal und sicher zu begleiten.
